# Exosomes: the key of sophisticated cell–cell communication and targeted metastasis in pancreatic cancer

**DOI:** 10.1186/s12964-021-00808-w

**Published:** 2022-01-15

**Authors:** Huan Zhang, Juan Xing, Zhujiang Dai, Daorong Wang, Dong Tang

**Affiliations:** 1 Clinical Medical College, Yangzhou University, Yangzhou, Jiangsu Province China; 2 Department of General Surgery, Institute of General Surgery, Northern Jiangsu People’s Hospital, Clinical Medical College, Yangzhou University, Yangzhou, 225001 China

**Keywords:** Pancreatic cancer (PaCa), Exosome, Cell–cell communication, Metastasis, Biomaker

## Abstract

Pancreatic cancer is one of the most common malignancies. Unfortunately, the lack of effective methods of treatment and diagnosis has led to poor prognosis coupled with a very high mortality rate. So far, the pathogenesis and progression mechanisms of pancreatic cancer have been poorly characterized. Exosomes are small vesicles secreted by most cells, contain lipids, proteins, and nucleic acids, and are involved in diverse functions such as intercellular communications, biological processes, and cell signaling. In pancreatic cancer, exosomes are enriched with multiple signaling molecules that mediate intercellular communication with control of immune suppression, mutual promotion between pancreas stellate cells and pancreatic cancer cells, and reprogramming of normal cells. In addition, exosomes can regulate the pancreatic cancer microenvironment and promote the growth and survival of pancreatic cancer. Exosomes can also build pre-metastatic micro-ecological niches and facilitate the targeting of pancreatic cancer. The ability of exosomes to load cargo and target allows them to be of great clinical value as a biomarker mediator for targeted drugs in pancreatic cancer.

**Video Abstract**

**Supplementary Information:**

The online version contains supplementary material available at 10.1186/s12964-021-00808-w.

## Background

Pancreatic cancer (PaCa) is the fourth leading cause of cancer mortality in the United States, with an overall 5-year survival rate of 5% to 15% in the United States. Although surgical resection is the only potentially curative therapy, only 20% of PaCa are eligible for surgical resection at the time of diagnosis due to the rapid progression of PaCa and lack of early diagnostic access [1, 2]. So, early diagnosis, prevention of metastasis, and effective treatment methods of pancreatic cancer are critical issues that need to be addressed [3]. The PaCa microenvironment, consisting of cancer cells, stromal cells, and extracellular matrix, is critical to the growth and survival of PaCa. The stromal cells that contribute to PaCa progression are mainly pancreatic stellate cells (PSCs), regulatory T cells (Tregs), myeloid-derived suppressor cells (MDSCs), and tumor-associated macrophages (TAMs). Besides, these cancerous cells can secrete extracellular components such as extracellular matrix (ECM), growth factors, and transforming growth factor-beta (TGFβ), thereby creating a microenvironment that supports the survival and progression of PaCa [4]. Exosomes are a pathway for the above cells to secrete extracellular components and transport metastatic signals, significantly impacting the PaCa microenvironment and the normal cell microenvironment.

Exosomes are extracellular vesicles 40–150 nm in diameter that all cells can release. They consist of a lipid bilayer surrounding a small cytoplasm without cellular organelles, allowing for local and systematic transfer of intercellular components [5]. Current studies have shown that exosomes can be synthesized by different types of cells and released into the extracellular environment, promoting PaCa proliferation, angiogenesis, invasion of adjacent normal tissue structures, distant metastasis, and the occurrence of chemotherapy resistance. Since the discovery of exosomes in 1983, the utility of exosomes in PaCa has become increasingly significant [6]. This review focuses on the function of exosomal transport signals of PaCa origin, mediation of intercellular communication, and regulation of PaCa metastasis. Due to their stability and accumulation in the circulatory system, exosomes are enriched for many signaling molecules, making them clinical biomarkers for PaCa. In addition, the ability of exosomes to act vectorially and bidirectionally have highlighted their clinical values in PaCa therapeutic research.

## Origin and structure of exosomes

**Fig. 1:**
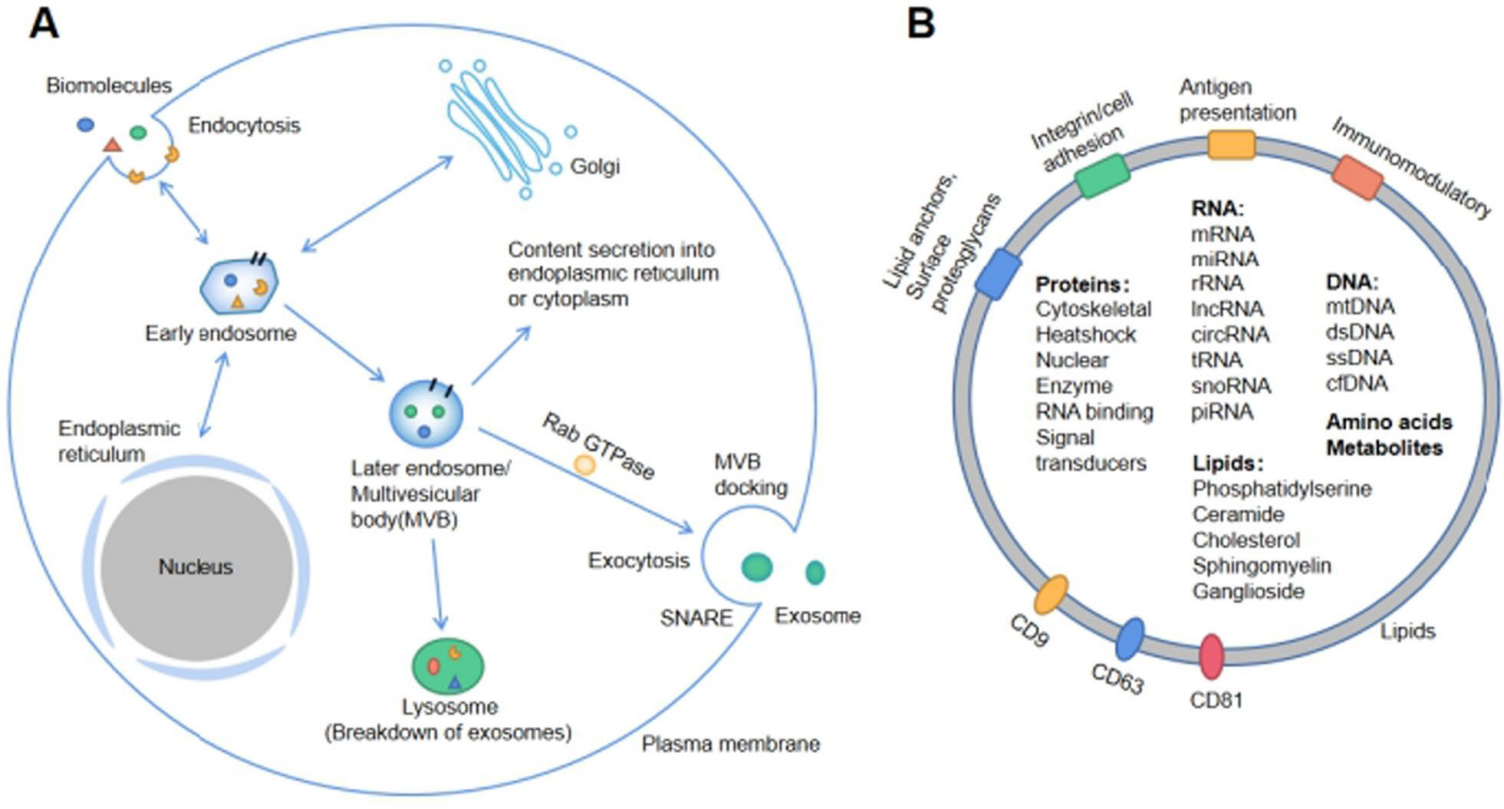
**A** Origin: Fluids and extracellular components enter cells, along with cell surface proteins, through endocytosis and plasma membrane fusion to form early endosomes. Early endosomes can form late endosomes (MVBs) through various mechanisms mediated by information exchange with intracellular materials such as Golgi and endoplasmic reticulum in the cytoplasm. Late endosomes exchange information with cytoplasm, are degraded by lysosomes or are mediated by Rab GTPase and SNARE proteins to reach the cell membrane to release exosomes. **B** Structure: Exosome surface proteins include integrins, immunomodulatory proteins, and so on. Exosomes contain different cell surface proteins, intracellular proteins, RNAs, DNAs, amino acids, and metabolites

Exosome production begins with endocytosis, followed by a complex intracellular pathway, ending with cytosolic exocytosis (Fig. 1A). Altered cancer genotypes, phenotypes, endocytosis characteristics, and increased plasma membrane remodeling may lead to endocytosis. It has been shown in previous studies that cancer cells secrete more exosomes compared to normal cells [5]. Early endosomes formed by embedding the plasma membrane can exchange and enrich signaling molecules with the cytoplasm, Golgi apparatus, and endoplasmic reticulum through mechanisms such as endosomal sorting complex required for transport (ESCRT), receptor-mediated internalization, and lipid interactions [7]. The "signaling endosome" determines the content and type of exosomal contents through cytoplasmic interactions [7]. The formation of later endosomes or multivesicular bodies (MVBs) from early endosomes has three known destinations: Firstly the direct secretion of contents into the endoplasmic reticulum or cytoplasm and the trans-Golgi network; secondly, phagocytosis and degradation by phagosomes, and finally fusion with the plasma membrane to secrete exosomes into the extracellular microenvironment (Fig. 1A) [8].

The secretion and release of exosomes into the extracellular microenvironment is dependent on several energetically active steps mediated by Rab GTPases, molecular motors, cytoskeletal proteins, and soluble N-ethylmaleimide-sensitive factor attachment protein receptors (SNAREs) [8, 9]. Previous studies demonstrated that plasmacytoma variant translocation 1 (PVT1) facilitates the transport of MVBs to the plasma membrane, thus promoting the secretion of PaCa-derived exosomes (Pex) [10]. The secreted exosomes can reach all body parts via the circulatory pathway and be taken up by specific organs, tissues, and cells. Exosomes can communicate with target cells through several known pathways: docking at the target cell's plasma membrane and activating intracellular signals through ligand-receptor interactions [8]. Another pathway is through fusion with the septum between the endocytic compartment through endocytosis and releasing its contents into the cytoplasm of the recipient cell, and lastly by absorption through membrane fusion and intracytoplasmic release.[8]

The lipid bilayer enveloping the exosome contains multiple signaling molecules, such as antigen-presenting molecules, integrins, surface proteoglycans, CD9, CD63, CD81, etc., which mediate exosome recognition and fusion, uptake and secretion (Fig. 1B). Exosomes carry a large number of cellular signaling molecules that regulate cell–cell communication, regulating the PaCa microenvironment in the proximity and later promoting PaCa invasion and metastasis. Exosomes can be modified to act as effective carriers for PaCa therapy and as a biomarker in PaCa liquid biopsies [8, 11, 12].

## Exosomes mediate cell–cell communication in PaCa

**Fig. 2:**
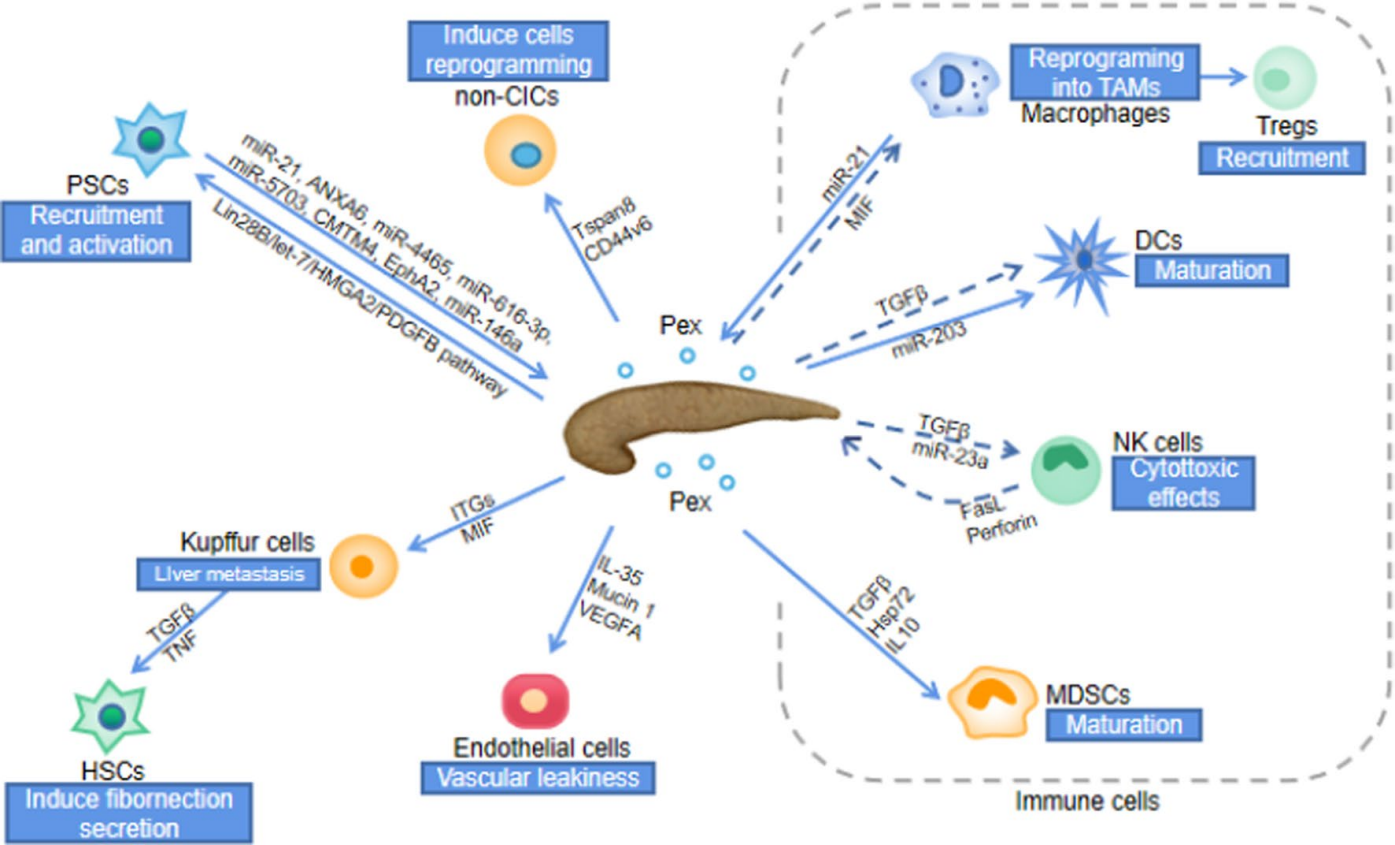
Pex mediates cell–cell communication. CICs lead to reprogramming of non-CICs by secreting Pex. The preferential fusion of Pex with Kupffer cells leads to the recruitment and activation of HSCs, the formation of a fibrotic microenvironment and ultimately to liver metastasis. PaCa activates PSCs by secreting exosomes, and activated PSCs promote PaCa proliferation, metastasis and drug resistance by secreting exosomes. In terms of immune cells, Pex leads to reprogramming of macrophages, recruitment of Tregs, inhibition of NK cells, maturation of MDSCs through signalling molecules and ultimately to immune suppression. (dashed lines: inhibit, solid lines: promote)

PaCa is not an isolated entity but a network system involving cellular communication between malignant and normal cells [8]. Currently, widely accepted modes of cell–cell communication include (1) chemoreceptor-mediated contact, (2) direct cell-to-cell contact, and (3) cell -cell synaptic contact [13]. On the one hand, Pex secreted by PaCa can transport nucleic acids, proteins, and lipids from donor cells to recipient cells, releasing signals that induce inflammatory responses, suppress immune responses, regulate the anti-apoptotic response of cancer cells and promote angiogenesis, thereby promoting cancer metastasis (Table 1); On the other hand, cells associated with PaCa, such as tumor-associated macrophages (TAMs), cancer-initiating cells (CICs) and pancreatic stellate cells (PSCs), which release exosomes, can promote the growth, drug resistance, metastasis and invasion of PaCa (Fig. 2) [8, 10, 11].

**Table 1:** Potential exosomes biomarkers of PaCa

Biomarkers	Exosome function	Signaling pathway	References
CD44v6	Enhance migration and invasion	Activate Wnt/β-Catenin pathway and increase PAI-1, MMP and TIM-1	[44, 95, 96]
Tspan8	Promote signaling, apoptosis-resistance, angiogenesis, EMT, motility and invasion	Increase expression of chemokine and receptor	[44, 97]
MIF	Promote the formation of the liver pre-metastatic niche	Up-regulate TGF-β expression, KC and HSC activation and induce fibronectin secretion	[32]
Clandin7	Promote migration and invasion	Increase pAkt/Bcl-2/Bcl-XL/MDR1, promote matrix degradation, and reprogram SC and HPC	[48]
Lin28B	Promote the recruitment of PSC	Activate Lin28B/let-7/HMGA2 /PDGFB axis	[52]
GPC1	Inform pancreas cancer burden	Carry mutant Kras mRNA and HS modifications	[98–100]
C1QBP	HSC activation and liver fibrosis	Induce phosphorylation of the IGF-1 signaling molecule	[97]
Plectin	Induce migration, proliferation and invasion	Not mentioned	[80]
miR-1246	Promote PSC proliferation and pancreatic fibrosis	Induce Akt/ERK activation and increase α-SMA and procollagen type I C-peptide	[51, 97, 101]
miR-301a-3p	Promote metastasis and EMT	Mediate M2 macrophage polarization via PTEN/PI3Kγ	[102]
SRSF1	Regulate exosome microRNA enrichment	Direct binding to miR-1246 sequence	[86]
lnc-Sox2ot	Promote EMT and stem cell like properties	Bind to the miR-200 family	[101]
ITGs	Promote tumor-specific metastasis	Promote adhesion and activate genes	[33]
AEP	Enhance invasion and promote metastasis	Regulate activation of PI3K/AKT signaling	[102]
CKAP4	Proliferation and migration of PDAC cells	DKK1-dependent endocytosis routes	[103]

### Pex-mediated immune suppression of PaCa

A necessary condition for the survival and development of PaCa cells is the capacity to evade host immune surveillance [14]. Pex promotes the escape of immune surveillance by inhibiting the activation and survival of lymphocytes, thereby inducing a loss of lymphocyte function [7, 15, 16].

T cells interact with Pex via the receptor/ligand signaling. In contrast, other lymphocytes (B cells, NK cells) and monocytes internalize Pex [17]. Pex can directly achieve immunosuppression by inducing apoptosis in activated anti-tumor effector cells or by polarising immune cells into a tumor-promoting phenotype. Pex can induce T cell apoptosis through pathways involving Fas ligand, TRAIL, and PDL-1 receptor-mediated, CD4^+^CD25^+^Foxp3^+^ Treg cells [18–20]. Moreover, Pex inhibits the proliferation of CD8^+^ T cells but promotes the expansion of CD4^+^ T cells, particularly Treg [17]. Pex increases the secretion of proinflammatory cytokines IL-1β, IL-6, and IL-10 while inhibiting the release of IFNγ, IL-2, and IL-17 from CD4^+^ and CD8^+^ T cells. This inhibits T cell proliferation and differentiation to Th1 and Th17 cells and promotes the production of Treg cells [8]. Macrophages are usually divided into two functional polarisation states: M1, which kills tumor cells, and M2, which promotes tumor growth [21]. It has been shown that exosomes modified by plasmid DNA expressing miR-155 or miR-125b-2 can lead to macrophage reprogramming in the pancreatic tumor microenvironment, leading to a potential pathway to inhibit PaCa invasion and metastasis [22]. In the tumor microenvironment, exosomes mediate the activation of macrophages into TAM, which exhibits M2 features [23]. TAM can further induce immune regulation, inflammatory responses, angiogenesis, and the establishment of a pre-metastatic microenvironment, thereby promoting tumor initiation and progression, invasion, and metastasis [24].

On the other hand, Pex can indirectly achieve immunosuppression by inhibiting functions necessary to maintain immunogenic responses such as activation, proliferation, and cytotoxicity. Pex deliver receptor-mediated signals to T cells that initiate sustained Ca^2+^ flux, resulting in subsequent activation of the relevant downstream pathways, alterations in the recipient cell transcriptome and ultimately translate into modified functional responses [17, 25]. In activated CD4^+^ T cells, STAT 5 phosphorylation is increased but is decreased in activated CD8^+^ T cells [15]. Adenosine generated from ATP by Pex converts activated B cells into regulatory B cells through CD39 and CD73, resulting in its inhibition [26]. Dendritic cells act as specialized antigen-presenting cells, influencing primary T cell response and achieving an anti-tumor cell response [27]. Pex can increase the levels of lncRNA and asparaginyl endopeptidase (AEP/legumain) mRNA in dendritic cells [28]. It can also indirectly amplify the proliferation of Treg and myeloid-derived suppressor cells (MDSCs) and upregulate their suppressive activity. This overall response contributes to tumor-induced immunosuppression and tumor immune escape [29, 30]. TAM induces an inflammatory response and is accepted by resident macrophages in the liver. High macrophage migration inhibitory factor (MIF) levels within exosomes of PaCa origin can induce upregulation of secreted factors through exosomal integrin αVβ5: TGFβ is associated with liver fibrosis. Furthermore, proinflammatory genes such as S100A8 and S100P are linked with metastasis [31–34]. It has been shown in previous studies that TAM-derived exosomal miR-501-3p can promote PaCa development through activation of the TGF-β signaling pathway and is a novel molecular target for PaCa immunotherapy [35]. In addition, exosomal miRNAs have been shown to induce immune tolerance and promote metastasis and invasion of tumor cells [36]. Enhancing immune activity and activating anti-tumor immune cells by isolating exosomal proteins and missing exosomal miRNAs also provides a pathway for PaCa treatment [37]. In short, Pex can mediate immune suppression indirectly or directly by inducing apoptosis of anti-tumor effector cells, inducing polarization of immune cells to a tumor-promoting phenotype with inhibition of the anti-tumor response.

### Reprogramming of non-CICs initiates metastasis potential of PaCa

Cancer-initiating cells (CICs) plays a vital role in the initiation of PaCa migration and metastasis [38, 39]. At the cellular level, CIC-derived exosomes can transfer certain features of CICs into non-cancer-initiating cells (non-CICs), thereby inducing their reprogramming and promoting their transformational features, such as non-anchored growth, apoptosis resistance, migration, and invasion, until they are phenotypically modified as CICs. At the level of PaCa, CICs induce tumor-stromal reorganization, stimulate angiogenesis, and promote immunosuppression of hematopoietic cells by producing exosomes resisting apoptosis. This results in a pre-metastatic ecological niche in the distal metastatic organ [11, 39, 40].

CIC-derived exosomes use CD44 variant isoform 6 (CD44v6) and Tspan8 as hubs to regulate miRNA production and function, leading to the reprogramming of non-CICs. Tspans belong to a family of 4 transmembrane proteins, consisting mainly of CD9, CD63, CD81, CD82, CD53, and CD37, and are 100 times more enriched in exosomes than in their source cells [41]. CD44v6 is Tspan8-associated in tetraspanin-enriched microdomains (TEM) biogenesis and targeting, and therefore promotes Tspan8 transcription [42–44]. Depending on cellular requirements, TEM may selectively recruit membrane-associated proteins such as integrins, proteases, and other relevant signaling molecules, thus acting as a specific signaling platform [41]. CICs-derived exosomes, acting as hub initiating non-CICs activation, is centrally shaped by CD44v6 and profit from message delivery by Tspan8 [42]. Upon uptake of PaCa-derived CD44v6-positive exosomes by other PaCa cells, they activate Wnt/β-Catenin signaling and upregulate the expression of plasminogen activator inhibitor 1, MMP, and tissue inhibitor of metalloproteases 1. This enhances PaCa cells migration and metastasis [44]. CD44v6 activity in PaCa relies on the association with receptor tyrosine kinases (RTK) and is engaged in Wnt signaling via associated LDL receptor related protein 6 (LRP6), which promotes catenin pathway activation [45–47]. Studies have demonstrated that RTK inhibitors can neutralize tumor progression by CICs-derived exosomes and may be an effective therapeutic approach to defeat CIC-derived exosomes [48]. PaCa exosomal Tspan8 may promote matrix degradation and reprogramming of the stroma and hematopoietic cells, which are essential steps for PaCa metastasis [11]. Blockade of exosome binding by anti-Tspan8 blockers inhibits CICs-derived exosomes in promoting PaCa progression and mitigates their deleterious effects on non-CICs [42]. In addition, the expression of Tspan8 and other CICs marker proteins, such as integrin α6β1, CD104, EpCAM, and CXCR4, is also mutually regulated in PaCa cells [11, 43]. So, with CD44v6 and Tspan8 acting as hubs, CICs-derived exosomes can induce non-CICs reprogramming, thereby inducing matrix reorganization and forming a cascade of pre-metastatic ecological for and initiating cancer metastasis.

### Pex mediate mutual promotion between PSCs and PaCa cells

Pancreas stellate cells (PSC, also known as cancer-associated fibroblasts (CAF)) plays a crucial role in the development of chronic pancreatitis, pancreatic fibrosis, and cancerous environment [49, 50]. Pex can induce PSC activation and pro-fibrosis, leading to a microenvironment of pre-cancerous fibrosis and the development of PaCa [51].

Pex can transfer Lin28B to PaCa cells, facilitating their recruitment and activation of PSCs via the Lin28B/let-7/HMGA2/PDGFB pathway [52]. Activation of PSC is characterized by the secretion of exosomes. Exosomal miRNAs are key messengers for communication between PSC and PaCa cells [53]. PSC-derived exosomal miR-21 promotes PaCa cell migration and EMT and enhances Ras/ERK and Ras/Akt signaling pathway activity in pancreatic ductal carcinoma cells [54]. PSC-derived exosomes can induce chemoresistance by transferring miR-21 into cancer cells, binding apoptosis peptidase-activating factor 1 (APAF1) or activating the phosphatidylinositol 3-kinase (PI3K)/Akt signaling pathway [55]. Downregulation of miR-21 was shown to inhibit the migration and invasion of PSC [56]. Besides, PSC-derived exosomes can also transmit chemoresistance by regulating the expression levels of EphA2, miR-146a and Snail signaling cascade in PaCa [57, 58]. Proliferation and metastasis of PaCa are promoted by regulating the expression levels of ANXA6, miR-4465, miR-616-3p, miR-5703 and CMTM4 [59–61]. PSCs contribute significantly to the chemoresistant nature of PaCa by producing extensive fibrous ECM, which results in (1) altered vasculature and decreased drug delivery to the tumor; (2) Reduced sensitivity to chemotherapeutic agents; and (3) increased epithelial-mesenchymal transition [62]. Hence, downregulation of exosomal miRNA or inhibition of exosomal secretion can inhibit the function of PSC, thus suppressing the occurrence, development and metastasis of PaCa. The exosomal inhibitor GW4869 inhibits the stimulatory effects of PSC on proliferation, migration and chemokine gene expression [63]. Pex is involved in PSC recruitment and activation, and activated PSC leads to proliferation, metastasis and chemoresistance of PaCa by secreting the exosome exosomal miRNAs. This bidirectional interaction between PSC and malignant cells, delivered by the exosome, favours tumour progression and metastasis [62].

## Exosomes induce targeted metastasis of PaCa

The extremely rapid progression, invasion and metastasis of pancreatic cancer leads to a very poor prognosis and high mortality rate. In addition to their impact on the local tumor microenvironment, exosomes also mediate distant cell–cell communication. They play an essential role in creating the pre-metastatic microenvironment of PaCa with invasion and metastasis through the regulation of fibroblast activation, ECM production, angiogenesis and immune surveillance.

**Fig. 3:**
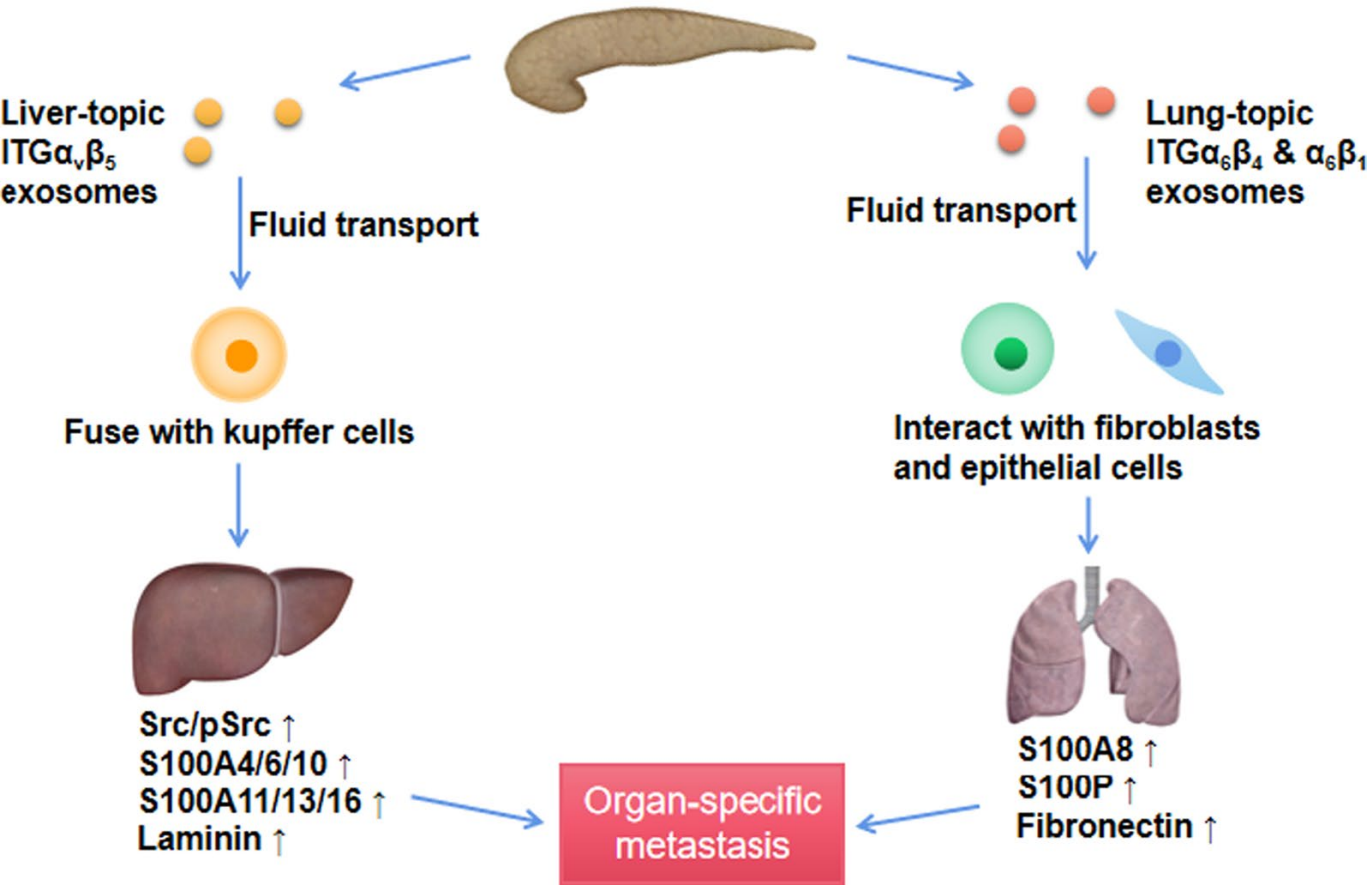
Pex direct organ-specific metastasis via integrins. Pex can display different integrin proteins on their surface; ITGα_6_β_4_- and ITGα_6_β_1_-expressing exosomes preferentially interact with fibroblasts and epithelial cells in lung, and ITGα_v_β_5_-expressing exosomes preferentially fuse with kupffer cells in liver. Once uptaken, Pex induce cellular changes in the target organ, thus promoting cancer cell colonization and organ-specific metastasis

Exosomes are critical determinants of organ-specific metastasis in PaCa through the expression levels of different integrins (ITG) [33, 64]. ITG can determine organotropic metastasis by fusing with organ-specific resident cells to establish pre-metastatic niche through activating Src phosphorylation and proinflammatory S100 expression [33, 65]. The organs most susceptible to metastasis from PaCa are the liver and lung. In PaCa liver metastasis, exosomes expressing ITGαvβ5 specifically bind to liver kupffer cells, Pex are transported to the liver via the humoral route fuses with kupffer cells (Fig. 3) [33]. The exosome-loaded MIF activates hepatic stellate cells, resulting in pro-fibrotic activity and remodeling of the extracellular matrix. Pro-fibrotic-related genes, such as TGFβ and TNF, are secreted by kupffer cells and increase liver metastasis burden [32]. Activated hepatic stellate cells participate in the inflammatory response, liver fibrosis and reconstruction of intrahepatic structures through proliferation and secretion of fibronectin. Intrahepatic sinusoidal pressure is increased through cellular contraction [32]. The resulting fibrotic microenvironment promotes the recruitment of bone marrow-derived cells that bind to fibronectin-rich hepatic sites, eventually forming pre-metastatic sites in the liver [32]. Furthermore, TAM acceptance by liver resident macrophages induces an inflammatory response, and high levels of MIF in the exocytotic body, mediated by ITGαVβ5, can induce upregulation of liver fibrosis-related factors and proinflammatory genes [32, 33]. In response to this inflammatory microenvironment, hepatic stellate cells are also activated, promoting liver metastasis and creating a vicious cycle. In order to access distant tissues, exosomes can disrupt endothelial cell junctions by increasing vascular permeability. Vesicles and cells enter the tissue parenchyma and alter the ECM, inducing thrombosis, creating a pre-metastatic microenvironment [66]. Increased VEGFA expression and serum concentrations in pancreatic cancer tumors are associated with worse prognosis and metastatic progression, particularly liver metastases [67]. In PaCa, exosomes can regulate VEGF expression via STAT3, NF-κb, and Mucin 1 [68–70]. Lung-tropic exosomes expressing ITGα6β4 or ITGα6β1 can specifically interact with S100A4-positive fibroblasts and surfactant protein C-positive epithelial cells and determining PaCa lung metastasis [33, 64]. ITGs significantly upregulated proinflammatory S100 gene expression and increased Src or phosphorylated Src levels in resident cells, thus promoting cancer cell colonization and organ-specific metastasis [65].

Furthermore, after homing to their target tissue, Pex can play a role in the activation of a reactive, myofibroblast-rich stroma that promotes a host of tumor-supportive processes such as ECM remodeling, proliferation, and angiogenesis [64, 71–74]. For example, exosomal-transduced TGFβ/Smad signaling has been shown to underline the differentiation process [64, 73]. Pex internalized by myofibroblast progenitors was shown to enhance their recruitment and trigger their differentiation into myofibroblast-like cells [64, 71–73]. By expressing immunomodulatory molecules such as CD39 and CD73, exosomes modulate host immunity, weakening immune effector cell responses and triggering immunosuppressive cells. This helps cancerous cells evade immune surveillance, allowing cancer progression and even generating a pre-metastatic microenvironment by activating an inflammatory response to promote completion [64].

In general, exosomes carry an abundance of pancreatic cancer signaling molecules closely associated with distant metastasis. These molecules determine the organ specificity of pancreatic cancer metastasis and contribute to establishing a pre-metastatic microenvironment.

## The clinical value of Pex: sources of biomarkers and drug carriers

Traditional biomarkers such as carcinoembryonic antigen (CEA) and cancer antigen 19–9 (CA19-9) have improved the diagnostic accuracy of PaCa, but they are less specific for PaCa [75]. Serous exosome concentrations tend to be higher in patients with PaCa than healthy controls. The load of exosomes varies according to the patient's health status, providing a powerful tool for PaCa biopsy [5]. A recent research hotspot in the field of exosome tools for PaCa research is surface-enhanced Raman scattering (SERS). Li TD et al. developed a PEARL SERS nano-tag that achieves ultra-sensitive and specific detection limits for Pex [75]. Pang Y et al. developed a DSN-assisted dual-SERS biosensor for direct quantification of target microRNAs in plasma-derived exosomes from pancreatic ductal adenocarcinoma (PDAC), chronic pancreatitis (CP) and normal controls (NC). They found significant SERS signal differences between these conditions [76]. In terms of cell surface markers, Pex can be enriched and carry many signaling molecules (Table 1). Glycosylation in cancer is considered a potential pathway for predicting cancer prognosis and exosomal glycan moieties are considered to be effective diagnostic markers in cancer detection [77, 78]. Glycan sequence analysis of exosomal subpopulations in PaCa cell lines and Pex detection techniques have been developed and reported for the study of exosomal glycan moieties.These techniques have also provided novel markers for PaCa [79, 80]. Fan J et al. isolated Pex from three PaCa cell lines that showed resistance to gemcitabine and reported that expression of exosome EphA2 could convey chemoresistance and predicted that it might be a powerful surface biomarker of exosomes [57, 81–83].

The ability of exosomes to be enriched with PaCa signaling molecules, mediate cell–cell communication, evade immune surveillance, bind specifically to target cells, and intermembranous transport show great promises as an area of research for drug delivery applications and therapeutic development [12, 84–86]. Kamerkar et al. show that exosomes from normal fibroblasts can be engineered to carry short interfering RNA or short hairpin RNA. These exosomes are more effective in targeting oncogenic kirsten rat sarcoma viral oncogene homolog (Kras) and inhibiting PaCa compared to liposomes [87]. McAndrews KM et al. show that engineered exosomes can serve as a delivery platform for CRISPR/Cas9 DNA to inhibit oncogenic Kras^G12D^ in vitro and suppress PaCa growth in vivo [88]. P21-activated kinase 4 (PAK4) is oncogenic when overexpressed, promoting cell survival, migration and unanchored growth [89, 90]. Using PAK4 as a therapeutic target in an in vivo mouse model with PaCa using exosome-mediated intra-tumor administration has been shown to reduce the growth of PaCa cells and improve survival rate in mice (*p* < 0.001) [91]. Pex exhibited the same efficacy and safety profile as polyethyleneimine in vivo RNAi transfection reagents, demonstrating their feasibility as drug carriers for use in PaCa therapy [91]. SRSF1 mediated the selective enrichment of exosomal miRNAs in PaCa cells by binding to the miR-1246 sequence [85]. GAIP The deletion of the C-terminus of the interacting protein could lead to the upregulation of the autophagy marker LC3II, autophagy in PaCa cells. It could also control the cellular transport pathway by regulating exosomes' secretion, which subsequently determines the loading of cellular cargo in exosomes [92]. What’s more, Zhou W et al. demonstrate an exosome-based dual delivery biosystem for enhancing PDAC immunotherapy as well as reversing tumor immunosuppression of M2-like tumor associated macrophages (M2-TAMs) upon disruption of galectin-9/dectin 1 axis [93]. The use of biomaterials, bone marrow mesenchymal stem cell exosomes, can significantly improve tumor targeting efficacy, thus increasing drug accumulation in the tumor site. Targeting of the tumor cells can also be improved by anchoring superparamagnetic nanoparticles to exosomes, which are “guided” towards the tumor by moderate magnetic fields [94].

In conclusion, a large number of studies have demonstrated that exosomes can be used as sources of biomarkers and drug carriers for PaCa treatment, and are of great value for pancreatic cancer diagnosis and treatment. However, the effectiveness of the above-mentioned markers and drug carrier functions is yet to be clinically validated. The complex biological properties of exosomes with selective loading mechanisms have not been thoroughly studied, which limits the progress of exosome research. How to achieve precise targeting of exosomes for gene editing is still an open question. Besides, we believe that targeting the metastatic signaling molecules on the surface of exosomes can make the target cells unable to recognize and accept exosomes, prevent the establishment of pre-metastatic microenvironment of pancreatic cancer and prevent invasive metastasis.

## Conclusion

Pex produced by MVBs secretion carries many signaling molecules for PaCa and are versatile and critical intercellular messengers. Between neighboring cells, exosomes help PaCa achieve immune evasion, promote angiogenesis and foster fibrosis in the pancreatic microenvironment, thus creating a cancer microenvironment that promotes the survival and development of PaCa. Between distal organ cells, exosomes are recognized and taken up by specific distal organ cells via humoral transport, creating a pre-metastatic ecological niche and stimulating distant metastasis. In the short 30 years since their discovery, exosomes have soared to the forefront of cell biology, cell signaling, cancer biology, immunology and drug delivery and have paved the way for clinical diagnostics and clinical therapeutic trials [7]. Exosomes are double-edged swords. If used by cancerous pancreatic cells, exosomes can shape the cancer microenvironment, creating a pre-metastatic ecological niche and facilitating its invasion and metastasis. If used therapeutically, exosomes can be a powerful aid to drug delivery and clinical treatment. The key, therefore, is how to use exosomes as a tool for our treatment. There are many unanswered questions about exosomes concerning their mode of transport, identification, and specific contents. The proper use of this therapeutic model requires further analysis and research of its specific clinical value.

## Data Availability

Not applicable.

## Abbreviations

AEP: Asparaginyl endopeptidase
CAFs: Cancer associated fibroblasts
CA19-9: Cancer antigen 19-9
CD44v6: CD44 variant isoform 6
CEA: Carcinoembryonic antigen
CICs: Cancer initiating cells
EMT: Epithelial-mesenchymal transition
ESCRT: Endosomal sorting complex required for transport
GPC1: Glypican-1
Kras: Kirsten rat sarcoma viral oncogene homolog
LRP6: LDL receptor related protein 6
MIF: Migration inhibitory factor
MVBs: Multivesicular bodies
non-CICs: Non-cancer-initiating cells
PaCa: Pancreatic cancer
PAK4: P21-activated kinase 4
PDAC: Pancreatic ductal adenocarcinoma
Pex: PaCa-derived exosomes
PSCs: Pancreatic stellate cells
RTK: Receptor tyrosine kinases
PVT1: Plasmacytoma variant translocation 1
SERS: Surface-enhanced Raman scattering
SNAREs: Soluble N-ethylmaleimide-sensitive factor attachment protein receptors
TAMs: Tumor-associated macrophages
TEM: Etraspanin-enriched microdomains
TGFβ: Transforming growth factor beta
